# Supplementary material for: Origin of fungal hybrids with pathogenic potential from warm seawater environments
Source: Nat Commun. 2023 Oct 30;14:6919. doi: 10.1038/s41467-023-42679-4 (PMC10616089; doi:10.1038/s41467-023-42679-4)
Supplement: Supplementary file 1 — Supplementary Information [file 41467_2023_42679_MOESM1_ESM.pdf]

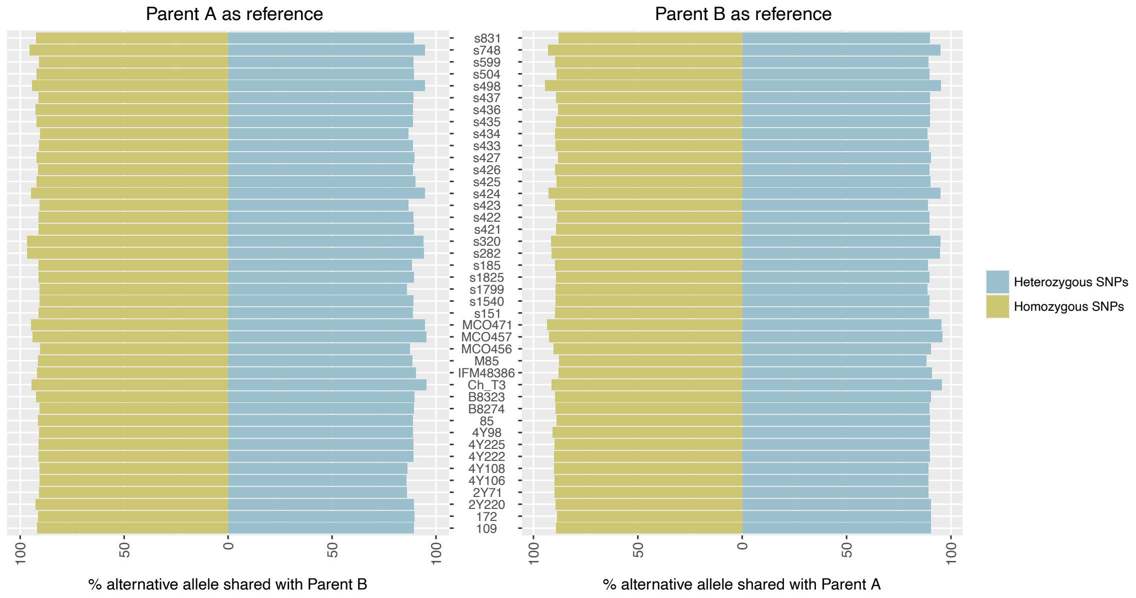

Supplementary Figure 1. **Alternative alleles in hybrid variants come either from parent A or parent B.** Bar plots showing the percentage of alternative alleles in hybrid variants shared with parent B when mapped to parent A (left panel) and vice-versa (right panel). Source data are provided as a Source Data file.

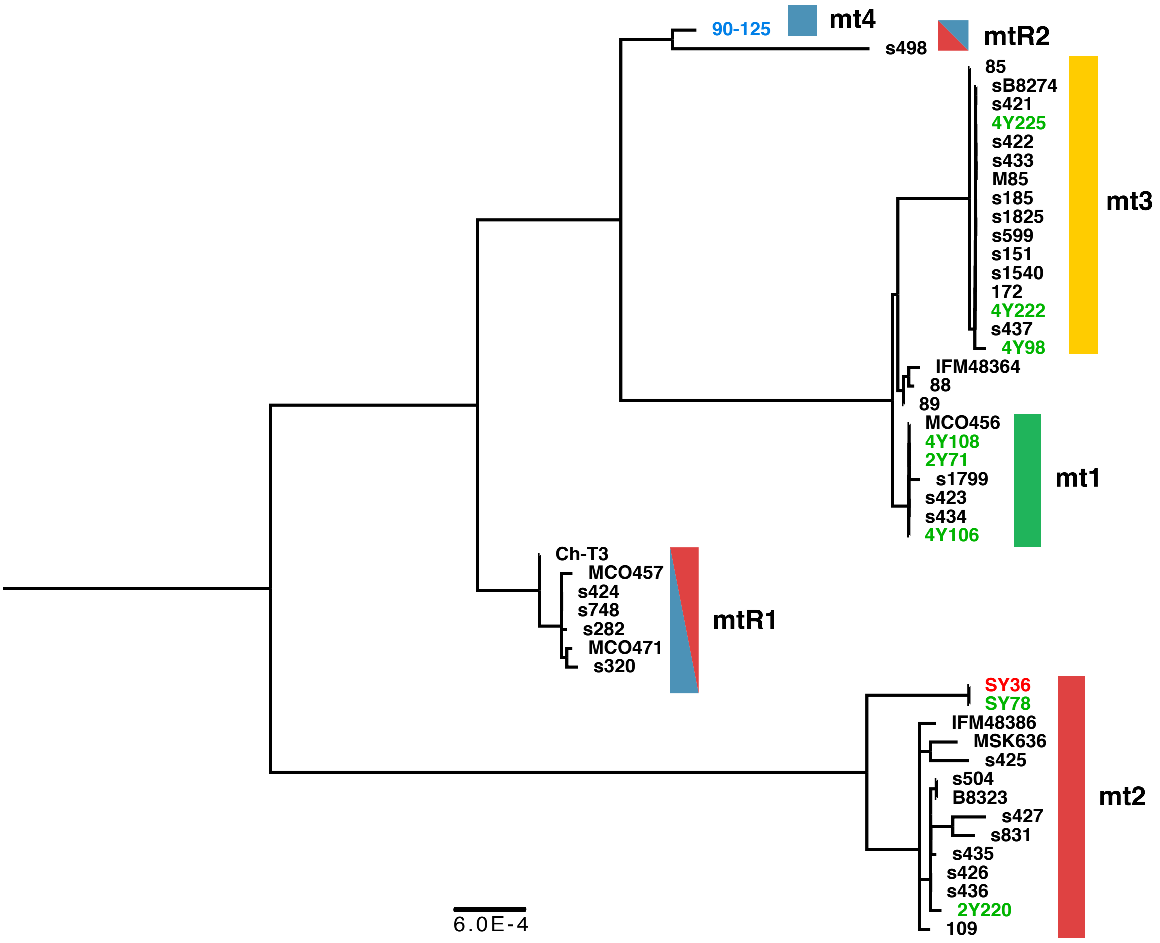

Supplementary Figure 2. Phylogenetic relationships between the mitochondrial genomes of *C. orthopsilosis* isolates. Maximum-likelihood tree based on variants of the mitochondrial genome of *C. orthopsilosis* strains. Single coloured bars represent different mitotypes (mt1-4) and multi coloured bars represent recombinant mitotypes (mtR1-2). Parent A Co90-125 is highlighted in blue, a representative strain of parent B SY36 in red and all other marine isolates in green. Source data are provided as a Source Data file.

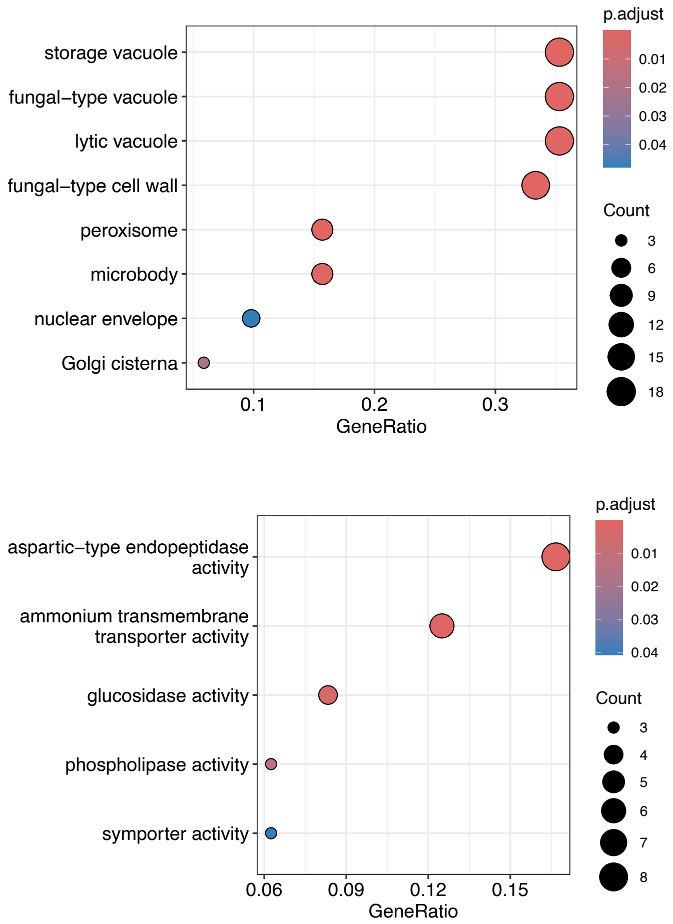

Supplementary Figure 3. GO term enrichment of Cell compartment (top) and Molecular function (bottom) terms of genes harboured in genomic regions that are parent B specific. GO term enrichment analysis was done using clusterProfiler v. 3.14.3, which performs the hypergeometric test. Source data are provided as a Source Data file.

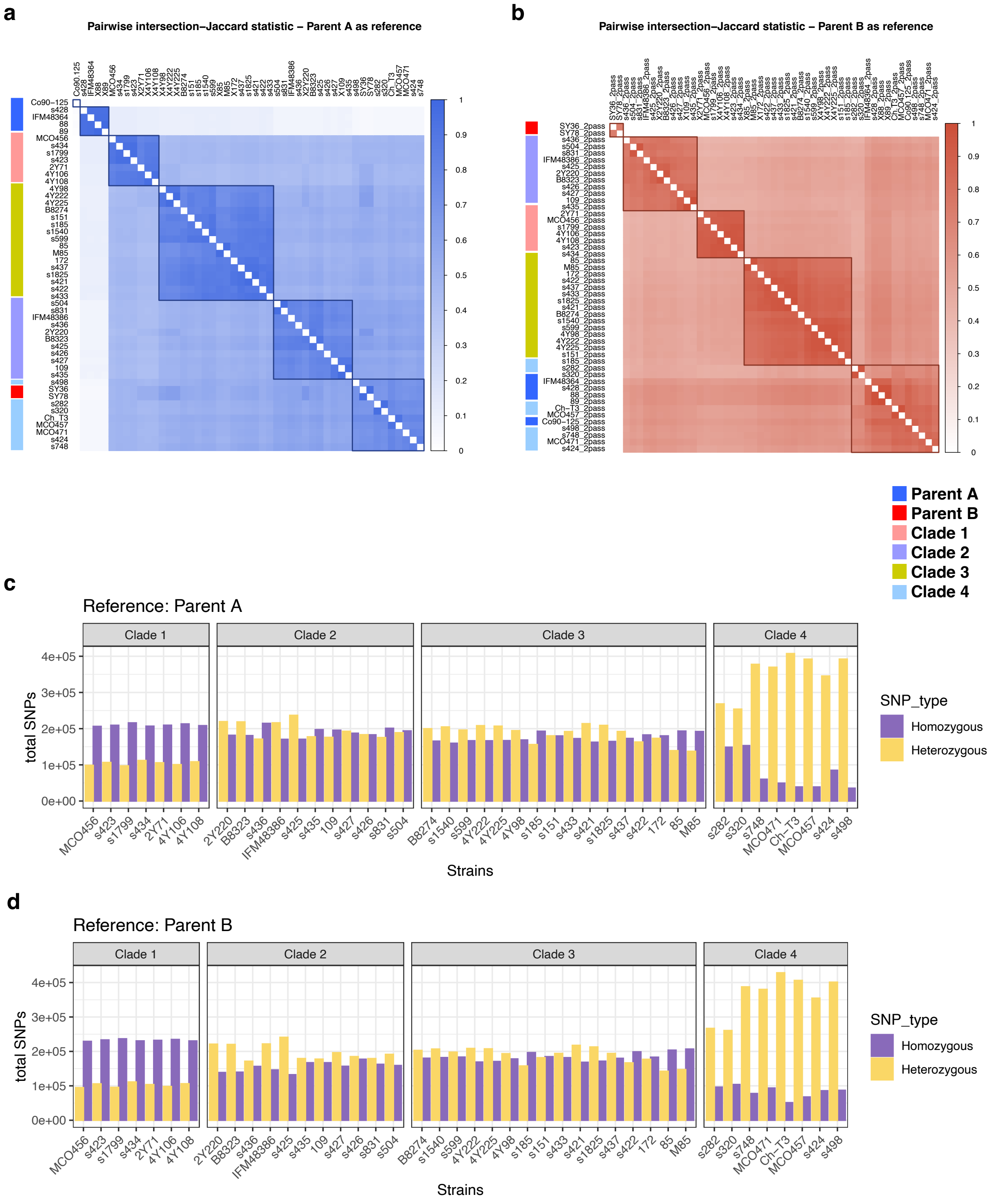

Supplementary Figure 4. Proportion and nature of variants differ significantly between *C. orthopsilosis* hybrid clades. (A-B) Jaccard similarity index based on variants taking either Co90-125 parent A (left) or SY36 parent B (right) as reference genome. Clades and parental lineages are indicated in

colour bars on the left side of the panels. **(C-D)** Total number of homozygous and heterozygous variants of each *C. orthopsilosis* hybrid strain mapped to parent A and parent B. Source data are provided as a Source Data file.

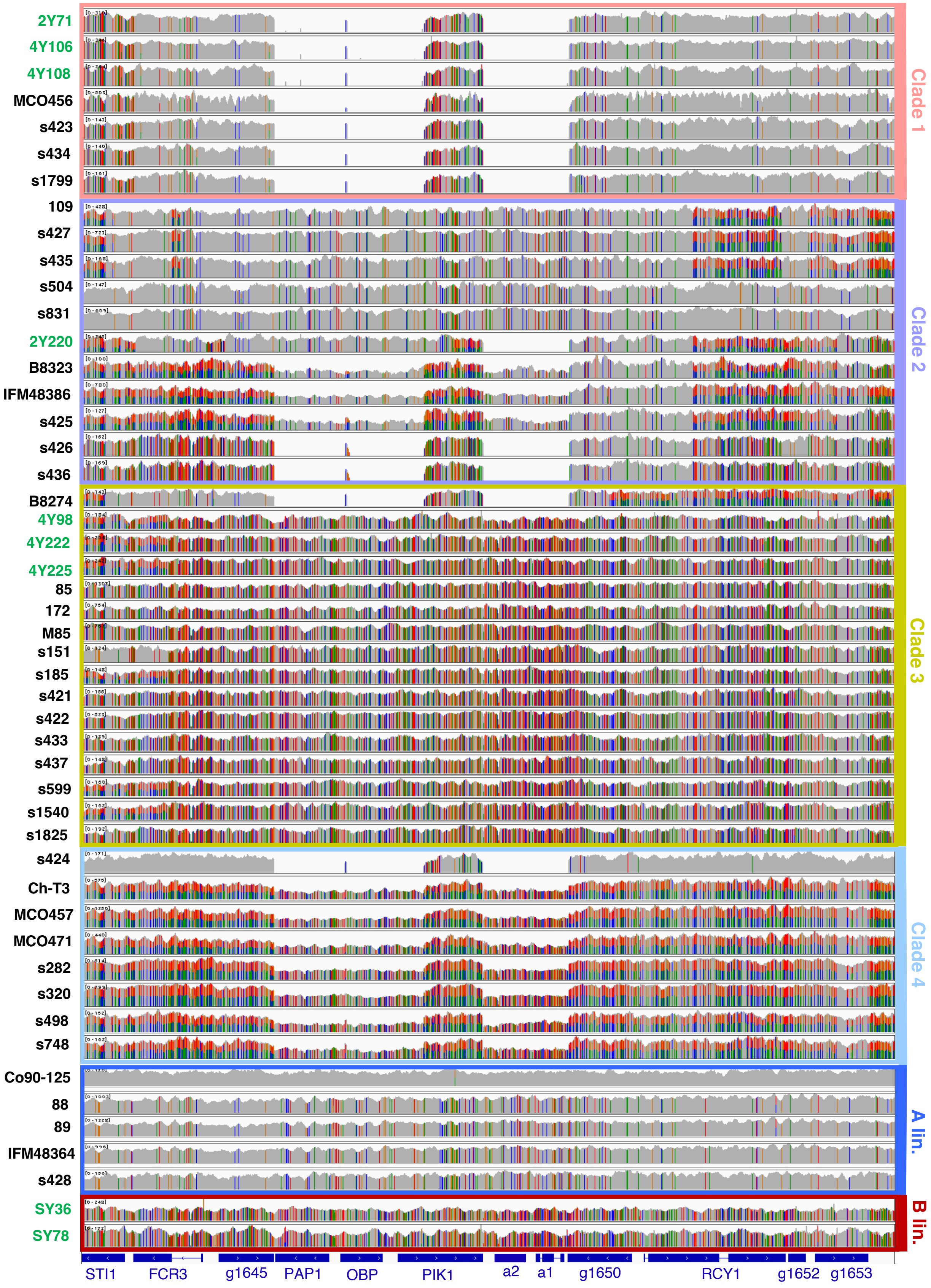

2Y71
4Y106
4Y108
MCO456
s423
s434
s1799
Clade 1
109
s427
s435
s504
s831
2Y220
B8323
IFM48386
s425
s426
s436
Clade 2
B8274
4Y98
4Y222
4Y225
85
172
M85
s151
s185
s421
s422
s433
s437
s599
s1540
s1825
Clade 3
s424
Ch-T3
MCO457
MCO471
s282
s320
s498
s748
Clade 4
Co90-125
88
89
IFM48364
s428
A lin.
SY36
SY78
B lin.
STI1
FCR3
g1645
PAP1
OBP
PIK1
a2
a1
g1650
RCY1
g1652
g1653

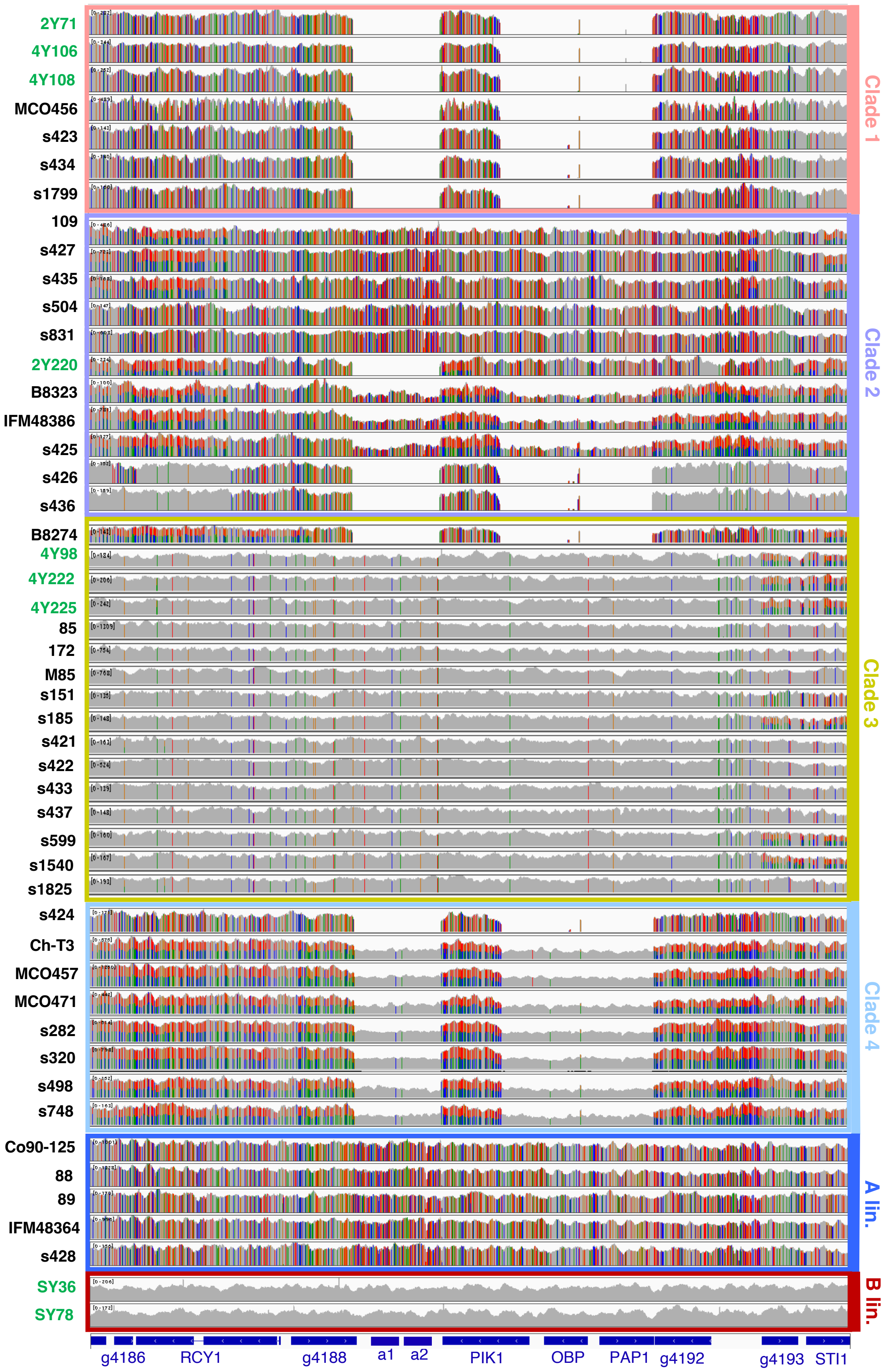
2Y71
4Y106
4Y108
MCO456
s423
s434
s1799
109
s427
s435
s504
s831
2Y220
B8323
IFM48386
s425
s426
s436
B8274
4Y98
4Y222
4Y225
85
172
M85
s151
s185
s421
s422
s433
s437
s599
s1540
s1825
s424
Ch-T3
MCO457
MCO471
s282
s320
s498
s748
Co90-125
88
89
IFM48364
s428
SY36
SY78
Clade 1
Clade 2
Clade 3
Clade 4
A lin.
B lin.
g4186
RCY1
g4188
a1
a2
PIK1
OBP
PAP1
g4192
g4193
STI1

Supplementary Figure 5. IGV tracks showing coverage and variants in the *MTL* of *C. orthopsilosis* strains mapped to parent A Co90-125 (top) or parent B SY36 (bottom). Clades and parental lineages are indicated in coloured rectangles. Marine isolates are labelled in green.

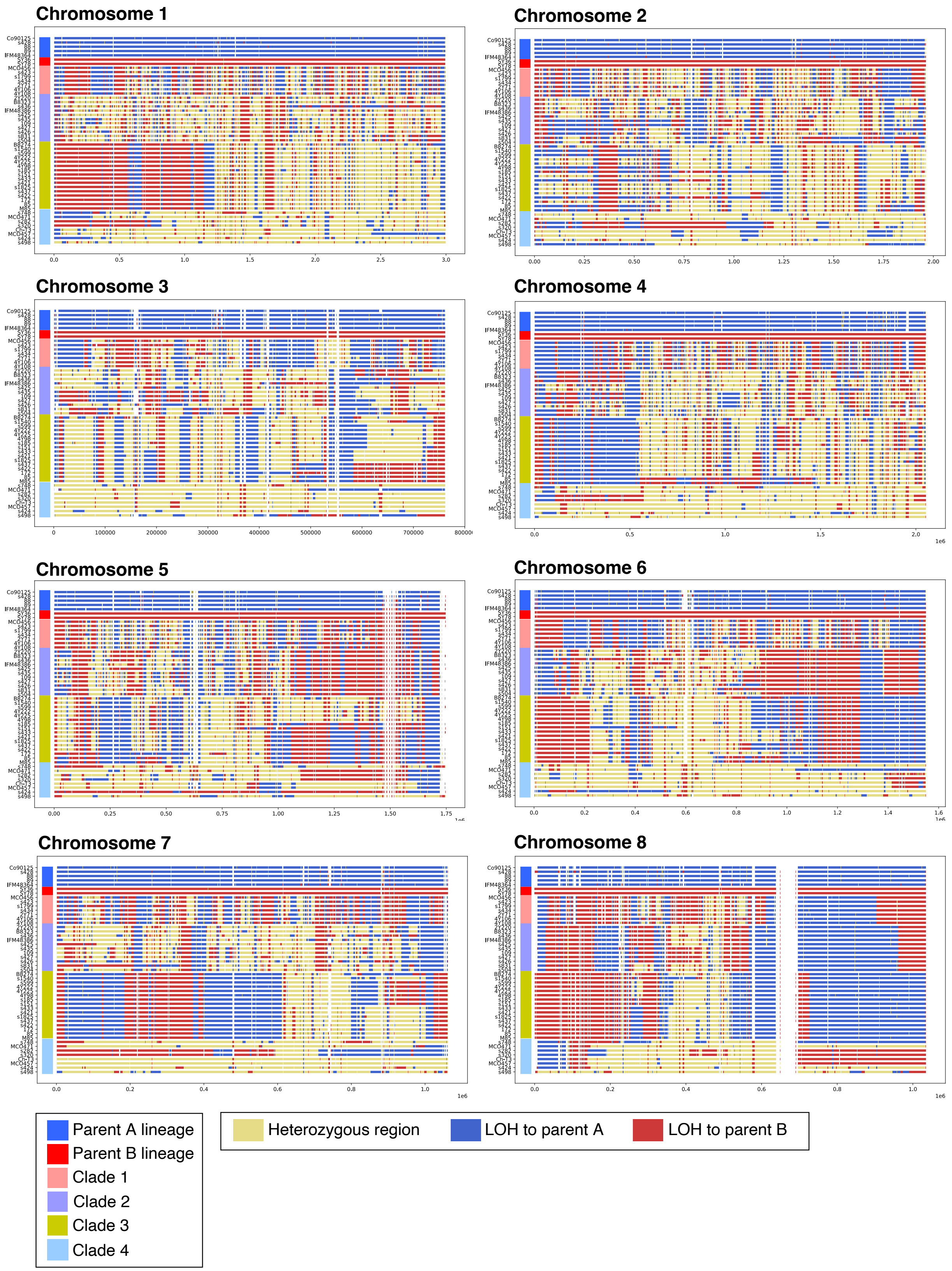
Chromosome 1
Chromosome 2
Chromosome 3
Chromosome 4
Chromosome 5
Chromosome 6
Chromosome 7
Chromosome 8
Parent A lineage
Parent B lineage
Clade 1
Clade 2
Clade 3
Clade 4
Heterozygous region
LOH to parent A
LOH to parent B

Supplementary Figure 6. Loss of heterozygosity across the genome of *C. orthopsilosis* strains. Blocks of LOH inherited from parent A (blue) or B (red) and regions of heterozygosity (yellow) are plotted along each chromosome for all *C. orthopsilosis* strains analysed in this study. Parent B was used as reference. Source data are provided as a Source Data file.

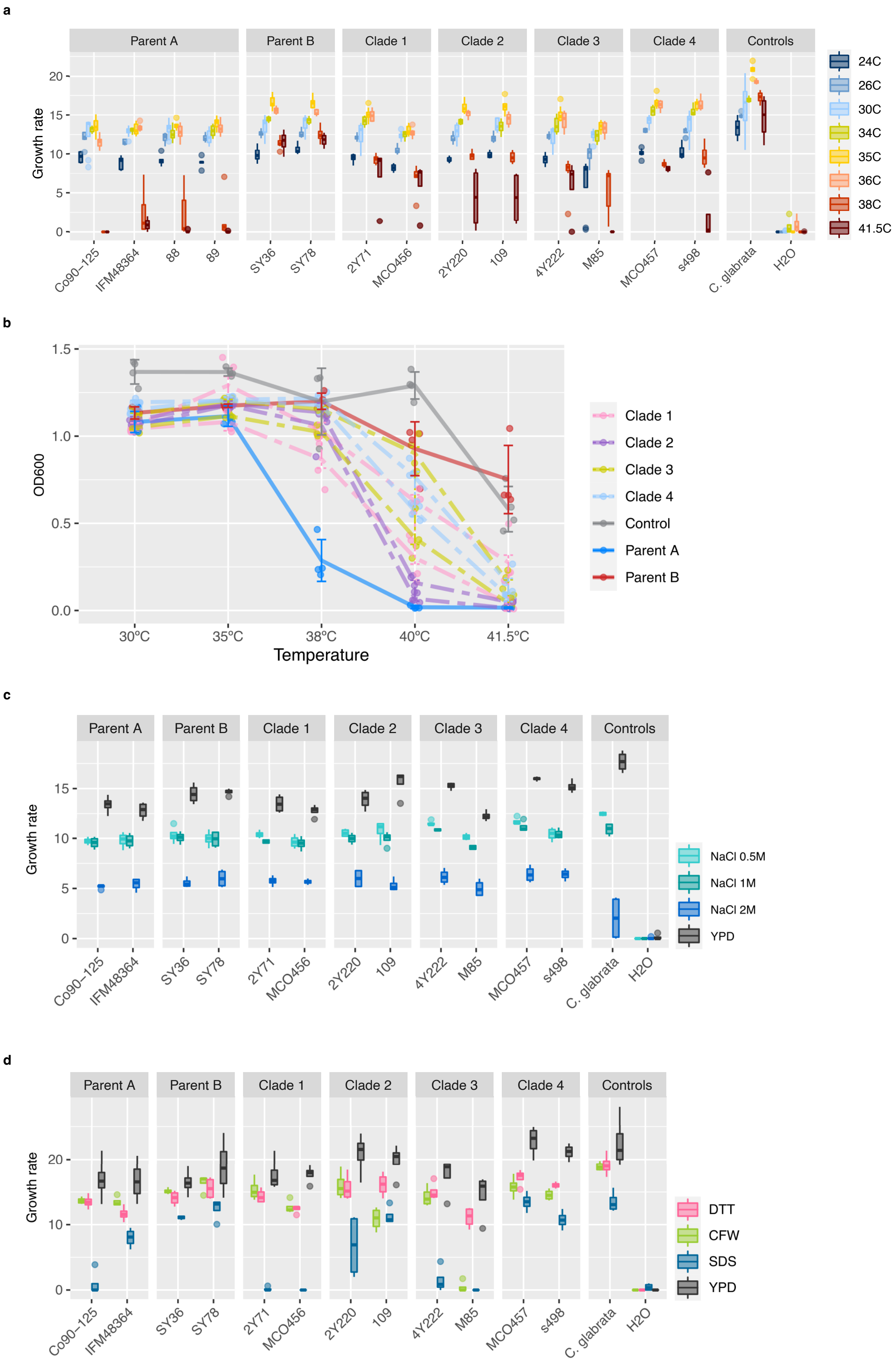
a
Parent A
Parent B
Clade 1
Clade 2
Clade 3
Clade 4
Controls
Growth rate
24C
26C
30C
34C
35C
36C
38C
41.5C
Co90−125
IFM48364
88
89
SY36
SY78
2Y71
MCO456
2Y220
109
4Y222
M85
MCO457
s498
C. glabrata
H2O
b
OD600
Clade 1
Clade 2
Clade 3
Clade 4
Control
Parent A
Parent B
30°C
35°C
38°C
40°C
41.5°C
Temperature
c
Growth rate
NaCl 0.5M
NaCl 1M
NaCl 2M
YPD
d
Growth rate
DTT
CFW
SDS
YPD

Supplementary Figure 7. Growth of *C. orthopsilosis* representative strains after 24h under different conditions (n = 4 biological replicates). **(A)** Growth rate of *C. orthopsilosis* isolates under temperatures ranging from 24 to 41.5ºC in solid medium. **(B)** Absorbance measurements of cultures containing *C. orthopsilosis* isolates in liquid medium grown at increasing temperatures. Data are presented as mean values +/- SD. **(C)** Growth rate of *C. orthopsilosis* isolates in the presence of different concentrations of NaCl in the medium (0.5M, 1M and 2M) and **(D)** in the presence of stressing agents 1,4-dithiothreitol (DTT), calcofluor-white (CFW) or sodium dodecyl sulphate (SDS) in solid medium. The centre line in boxplots indicates the median value, the boxes contain the Q1 and Q3 quartiles (IQR). The whiskers extend up to 1.5 × IQR. Source data are provided as a Source Data file.

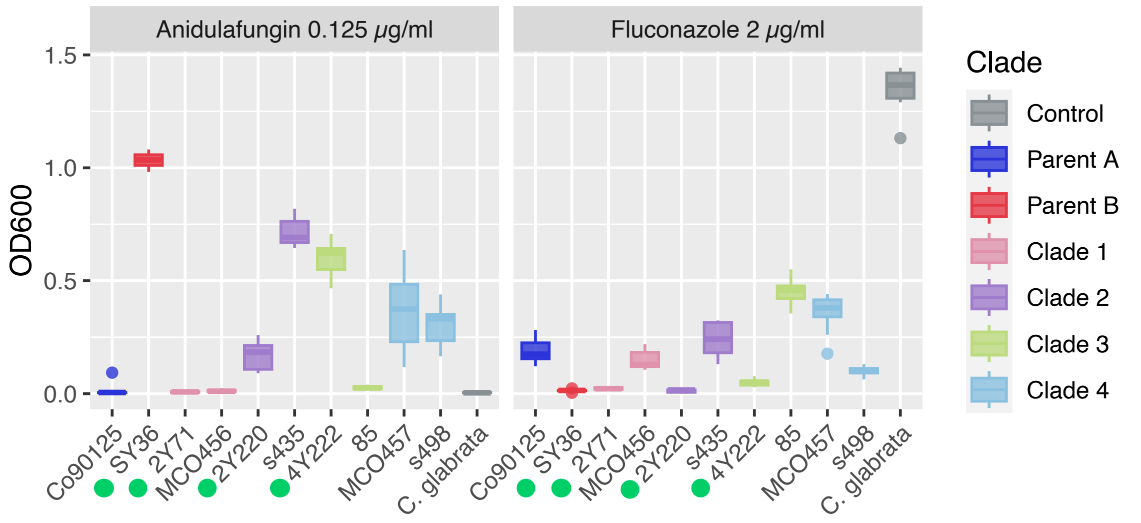

Supplementary Figure 8. Absorbance measurements of cultures containing *C. orthopsilosis* isolates (n = 4 biological replicates) in liquid medium grown for 24h at 30ºC in the presence of either 0.125µg/ml of anidulafungin (left panel) or 2µg/ml of fluconazole (right panel). Green circles indicate environmental isolates. The centre line in boxplots indicates the median value, the boxes contain the Q1 and Q3 quartiles (IQR). The whiskers extend up to 1.5 × IQR. Source data are provided as a Source Data file.

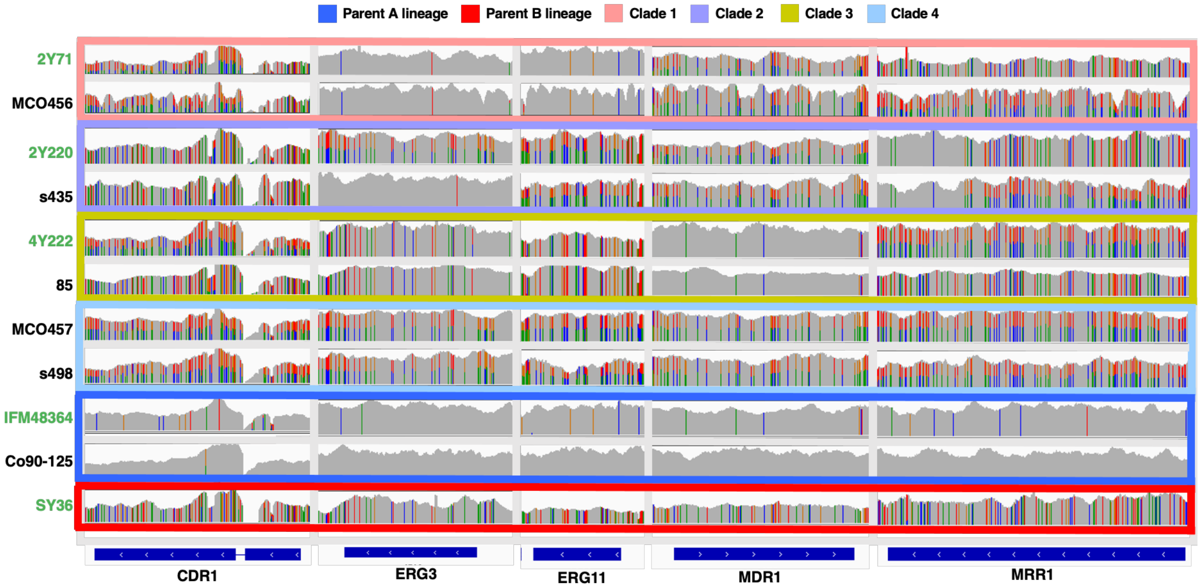

Supplementary Figure 9. Coverage tracks showing homozygous and heterozygous variants within genes related to fluconazole resistance. Sequencing reads were mapped to the reference genome of parent A. Strains susceptible to fluconazole are highlighted in green.

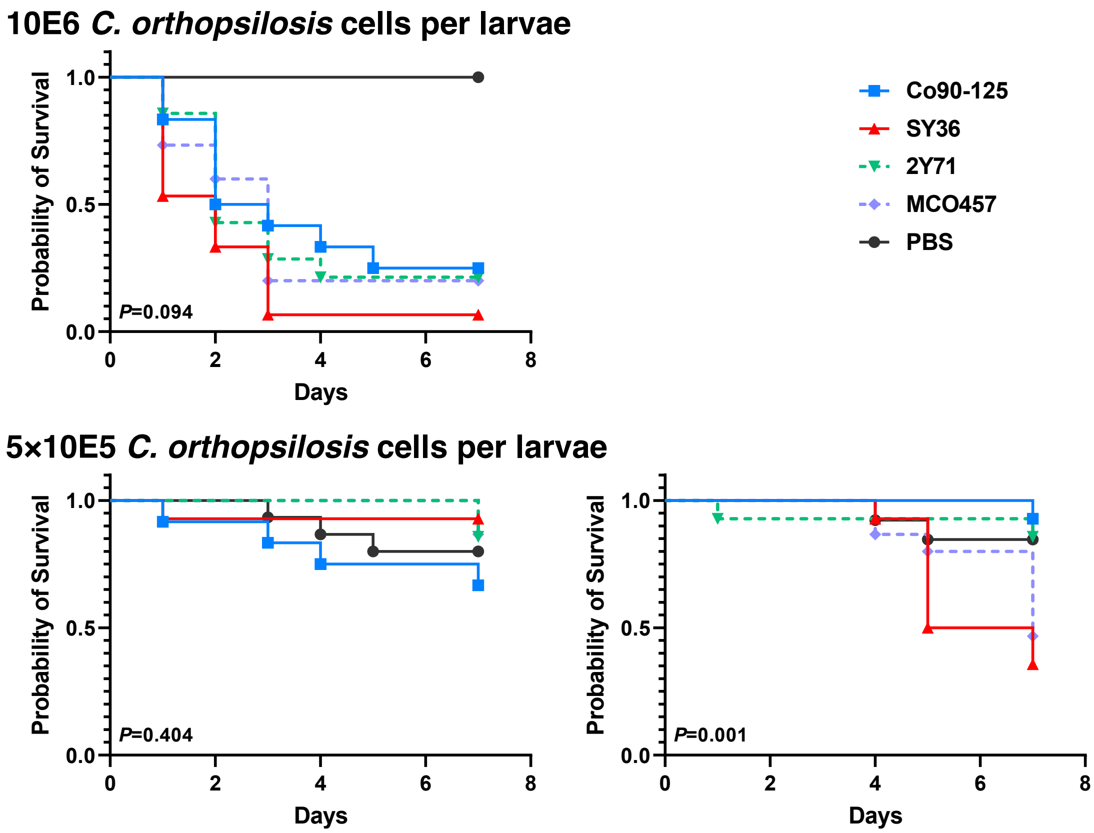

Supplementary Figure 10. Survival curves of *G. mellonella* infected with 10^6 (top) or $5x10^5$ (bottom) *C. orthopsilosis* cells per larva during a period of 7 days. *P*-values calculated by the log rank (Mantel-Cox) test are indicated. Source Data are provided as a Source Data file.

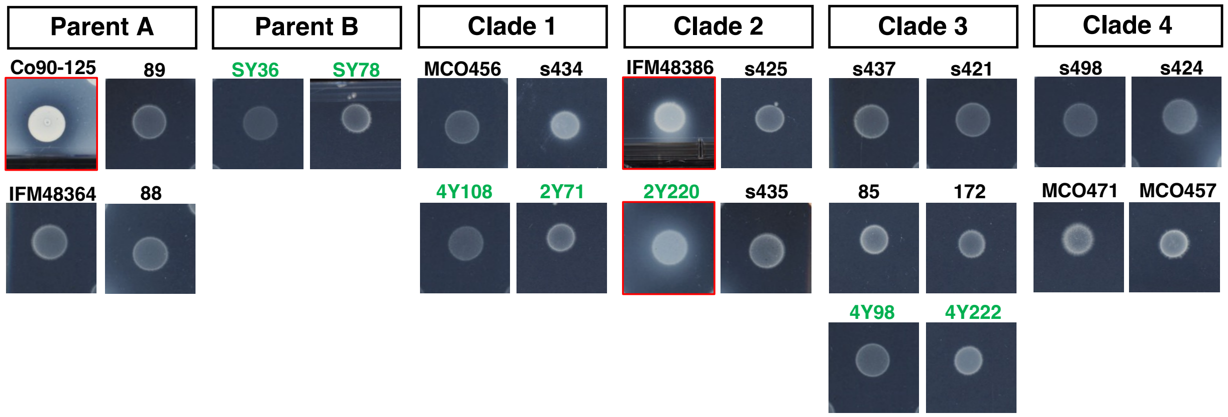

Supplementary Figure 11. Secretion of aspartyl proteases by *C. orthopsilosis* parental and hybrid strains. Environmental strains are highlighted in green writing. Colonies showing secretion of aspartyl proteases are highlighted with a red square. Source data are provided as a Source Data file.

Supplementary Table 1. GO term enrichment analysis of genes harboured in LOH blocks inherited from parent B shared by all strains belonging to clade 4. Source data are provided as a Source Data file.

Term category: **molecular function**				
overlist	term	term level	adj.pvalue	term name
**1**	GO:0004190	**1**	2.240720e-05	aspartic-type endopeptidase activity
**1**	GO:0070001	**1**	2.240720e-05	aspartic-type peptidase activity
Term category: **cellular component**				
overlist	term	term level	adj.pvalue	term name
**1**	GO:0005576	**1**	1.356900e-07	extracellular region
**1**	GO:0005618	**1**	1.866900e-04	cell wall
**1**	GO:0009277	**1**	1.866900e-04	fungal-type cell wall
**1**	GO:0009897	**1**	4.042750e-07	external side of plasma membrane
**1**	GO:0009986	**1**	2.403130e-05	cell surface
**1**	GO:0030312	**1**	1.866900e-04	external encapsulating structure
**1**	GO:0031224	**1**	9.379040e-04	intrinsic component of membrane
**1**	GO:0031225	**1**	7.794550e-07	anchored component of membrane
**1**	GO:0031226	**1**	2.240720e-05	intrinsic component of plasma membrane
**1**	GO:0031233	**1**	2.633360e-07	intrinsic component of external side of plasma membrane
**1**	GO:0031362	**1**	2.633360e-07	anchored component of external side of plasma membrane
**1**	GO:0044459	**1**	3.458700e-04	plasma membrane part
**1**	GO:0046658	**1**	7.794550e-07	anchored component of plasma membrane
**1**	GO:0098552	**1**	2.240720e-05	side of membrane
**2**	GO:0044422	**1**	2.240720e-05	organelle part
**2**	GO:0044446	**1**	2.240720e-05	intracellular organelle part
