# Supplementary material for: Origin of fungal hybrids with pathogenic potential from warm seawater environments
Source: Nat Commun. 2023 Oct 30;14:6919. doi: 10.1038/s41467-023-42679-4 (PMC10616089; doi:10.1038/s41467-023-42679-4)
Supplement: Supplementary file 3 — Description of Additional Supplementary Files [file 41467_2023_42679_MOESM3_ESM.pdf]

Description of Additional Supplementary Files:

Supplementary Data 1: Isolation source and conditions of C. orthopsilosis marine strains

Supplementary Data 2: Summary of mapping and variant calling stats of C. orthopsilosis marine isolates mapped to parent A Co90-125.

Supplementary Data 3: Overview of shared SNPs between C. orthopsilosis hybrids and SY36 parental B strain.

Supplementary Data 4: Summary of mapping and variant calling stats of C. orthopsilosis strains mapped to parent B SY36.

Supplementary Data 5: Summary loss of heterozygosity events detected in C. orthopsilosis strains.

Supplementary Data 6: Summary of copy number variants (CNV) C. orthopsilosis parental and hybrid strains.
