# Supplementary material for: Origin of fungal hybrids with pathogenic potential from warm seawater environments
Source: Nat Commun. 2023 Oct 30;14:6919. doi: 10.1038/s41467-023-42679-4 (PMC10616089; doi:10.1038/s41467-023-42679-4)
Supplement: Supplementary file 10 — Reporting Summary [file 41467_2023_42679_MOESM10_ESM.pdf]

nature portfolio

Corresponding author(s): Toni Gabaldón

Last updated by author(s): 2023/09/05

Reporting Summary

Nature Portfolio wishes to improve the reproducibility of the work that we publish. This form provides structure for consistency and transparency in reporting. For further information on Nature Portfolio policies, see our Editorial Policies and the Editorial Policy Checklist.

Statistics

For all statistical analyses, confirm that the following items are present in the figure legend, table legend, main text, or Methods section.

n/a	Confirmed	
☐	☒	The exact sample size (*n*) for each experimental group/condition, given as a discrete number and unit of measurement
☐	☒	A statement on whether measurements were taken from distinct samples or whether the same sample was measured repeatedly
☐	☒	The statistical test(s) used AND whether they are one- or two-sided<br>*Only common tests should be described solely by name; describe more complex techniques in the Methods section.*
☐	☒	A description of all covariates tested
☐	☒	A description of any assumptions or corrections, such as tests of normality and adjustment for multiple comparisons
☐	☒	A full description of the statistical parameters including central tendency (e.g. means) or other basic estimates (e.g. regression coefficient) AND variation (e.g. standard deviation) or associated estimates of uncertainty (e.g. confidence intervals)
☐	☒	For null hypothesis testing, the test statistic (e.g. F, t, r) with confidence intervals, effect sizes, degrees of freedom and P value noted<br>*Give P values as exact values whenever suitable.*
☐	☒	For Bayesian analysis, information on the choice of priors and Markov chain Monte Carlo settings
☐	☒	For hierarchical and complex designs, identification of the appropriate level for tests and full reporting of outcomes
☒	☐	Estimates of effect sizes (e.g. Cohen's d, Pearson's r), indicating how they were calculated

Our web collection on statistics for biologists contains articles on many of the points above.

Software and code

Policy information about availability of computer code

Data collection	no software was used

Data analysis	Raw sequencing data analysis: FastQC v0.11.9 (http://www.bioinformatics.babraham.ac.uk/projects/fastqc/), Trimmomatic v0.39, KAT v2.4.2, NanoFilt v2.7.1, Porechop v0.2.3,<br><br>Read mapping, small variant calling and detection of CNVs: BWA-MEM v0.7.17, GATK v4.1.9.0, SAMtools v1.9, IGV v2.8.13, nQuire (commit a990a88ef14b38f257f1a0d368ba8be1bd3d7e4b), PerSVade v.1.02.4<br><br>Genome assembly and genome analysis: Pilon v1.22, Augustus v3.2.3, BUSCO v4.1.4, Platanus v1.2.4, Canu v1.8, DBG2OLC v20180222, WTDBG2 v2.1, Ragout v2.2, Quast v5.0.2, Last v2.31.1, clusterProfiler v. 3.14.3<br><br>Phylogeny: Bedtools v2.30.0, IQTree v2.0.3, FigTree v1.4.4, Splitstree v5.2.25, JLOH v0.15.1, HaploTypo v1.0.1, MAFFT v7.475, TrimAl v1.4.rev15, PhylomeDB v5<br><br>Phenotypic analyses were done using Q-PHAST, a tool which has not yet been published although a manuscript is in preparation. The tool involves image processing of colony samples and calculation of growth rates and Area Under the Curve (AUC) which were carried out with an in-house pipeline (https://github.com/Gabaldonlab/imageAnalysisPipeline_solid96wellPlates) based on the software Colonyzer (Lawless, Wilkinson, Young, Addinall & Lydall. Colonyzer: Automated quantification of micro-organism growth characteristics on solid agar. BMC Bioinformatics 11, 1–12 (2010).)

For manuscripts utilizing custom algorithms or software that are central to the research but not yet described in published literature, software must be made available to editors and reviewers. We strongly encourage code deposition in a community repository (e.g. GitHub). See the Nature Portfolio guidelines for submitting code & software for further information.

Data

Policy information about availability of data

All manuscripts must include a data availability statement. This statement should provide the following information, where applicable:

- Accession codes, unique identifiers, or web links for publicly available datasets
- A description of any restrictions on data availability
- For clinical datasets or third party data, please ensure that the statement adheres to our policy

The whole genome sequencing data generated in this study has been deposited in the NCBI sequence read archive (SRA) database under accession code PRJNA767198 [https://www.ncbi.nlm.nih.gov/bioproject/PRJNA767198]. All C. orthopsilosis raw sequencing Illumina reads are under BioSample accessions SAMN21907760-68 [https://www.ncbi.nlm.nih.gov/biosample/SAMN21907760/ https://www.ncbi.nlm.nih.gov/biosample/SAMN21907761/ https://www.ncbi.nlm.nih.gov/biosample/SAMN21907762/ https://www.ncbi.nlm.nih.gov/biosample/SAMN21907763/ https://www.ncbi.nlm.nih.gov/biosample/SAMN21907764/ https://www.ncbi.nlm.nih.gov/biosample/SAMN21907765/ https://www.ncbi.nlm.nih.gov/biosample/SAMN21907766/ https://www.ncbi.nlm.nih.gov/biosample/SAMN21907767/ https://www.ncbi.nlm.nih.gov/biosample/SAMN21907768/]. C. orthopsilosis ONT data is under BioSample accession SAMN21909361 [https://www.ncbi.nlm.nih.gov/biosample/SAMN21909361]. Genome assembly of C. orthopsilosis parent B strain SY36 ASM2665022v1 is under BioSample accession SAMN21909361 [https://www.ncbi.nlm.nih.gov/datasets/genome/GCA_026650225.1/]. Additional sequencing libraries used in this study were retrieved from BioProjects PRJEB4430 https://www.ncbi.nlm.nih.gov/bioproject/PRJEB4430], PRJNA322245 [https://www.ncbi.nlm.nih.gov/bioproject/PRJNA322245] and PRJNA520893 [https://www.ncbi.nlm.nih.gov/bioproject/PRJNA520893]
Ortholog sequences are available in the PhylomeDB v5 database [http://phylomedb.org].

Research involving human participants, their data, or biological material

Policy information about studies with human participants or human data. See also policy information about sex, gender (identity/presentation), and sexual orientation and race, ethnicity and racism.

Reporting on sex and gender	This information was not collected as out research did not involve any human participants
Reporting on race, ethnicity, or other socially relevant groupings	Not applicable
Population characteristics	Not applicable
Recruitment	Not applicable
Ethics oversight	Not applicable

Note that full information on the approval of the study protocol must also be provided in the manuscript.

Field-specific reporting

Please select the one below that is the best fit for your research. If you are not sure, read the appropriate sections before making your selection.

☒ Life sciences ☐ Behavioural & social sciences ☐ Ecological, evolutionary & environmental sciences

For a reference copy of the document with all sections, see nature.com/documents/nr-reporting-summary-flat.pdf

Life sciences study design

All studies must disclose on these points even when the disclosure is negative.

Sample size	In this study, the genomes of C. orthopsilosis isolates were studied. We analysed the genomes of all available clinical isolates and seven newly described marine strains. The complete dataset comprised 49 C. orthopsilosis strains.
Data exclusions	No data were excluded from the analyses.
Replication	In our phenotype analyses in solid medium involving drugs and stressing agents and in experiments performed in liquid medium, we performed one experiment using four biological replicates of each strain.<br>In our phenotype analyses in solid medium involving different temperatures we performed two independent experiments using four biological replicates of each strain in each experiment.<br>All attempts at replication were successful and are reported in our manuscript.<br>For the virulence assays, we performed four independent experiments using two biological replicates of each tested strain. All attempts at replication were successful and are reported in our manuscript.
Randomization	Randomisation was not relevant to this study as all strains were subject to the same antifungal drug treatment and conditions.
Blinding	Blinding was not relevant to this study as it was considered unnecessary and because all strains were subject to the same antifungal drug treatment and conditions.

Reporting for specific materials, systems and methods

We require information from authors about some types of materials, experimental systems and methods used in many studies. Here, indicate whether each material, system or method listed is relevant to your study. If you are not sure if a list item applies to your research, read the appropriate section before selecting a response.

Materials & experimental systems

n/a	Involved in the study
☒	☐ Antibodies
☒	☐ Eukaryotic cell lines
☒	☐ Palaeontology and archaeology
☐	☒ Animals and other organisms
☒	☐ Clinical data
☒	☐ Dual use research of concern
☒	☐ Plants

Methods

n/a	Involved in the study
☒	☐ ChIP-seq
☒	☐ Flow cytometry
☒	☐ MRI-based neuroimaging

Animals and other research organisms

Policy information about studies involving animals; ARRIVE guidelines recommended for reporting animal research, and Sex and Gender in Research

Laboratory animals	Virulence experiments involved the use of Galleria mellonella larvae. The Galleria mellonella, were originally provided by de Jong, A. W., van Veldhuizen, D., Groot, A. T. & Hagen, F. Standardized methods to rear high-quality Galleria mellonella larvae for the study of fungal pathogens. Entomol. Exp. Appl. 170, 1073–1080 (2022) and then reared in our laboratory for two generations before the first experiment. The larvae were two weeks of age.
Wild animals	No wild animals were used in the study
Reporting on sex	This information was not collected
Field-collected samples	No field collected samples were used in the study
Ethics oversight	No ethical approval is required for studies involving Galleria mellonella

Note that full information on the approval of the study protocol must also be provided in the manuscript.
